# Effectiveness of Hydrogen Peroxide Nebulization Combined with Manual Cleaning in Reducing Surface-Associated Microorganisms in an Indoor Fitness Environment

**DOI:** 10.3390/microorganisms14081821

**Published:** 2026-08-18

**Authors:** Alexander Martens, Mohamad Motevalli, Markus Schauer, Susanne Mair, Brigitte König

**Affiliations:** 1 Institute for Medical Microbiology and Virology, University of Leipzig Medical Center, 04103 Leipzig, Germany; 2 Department of Sport Science, German University of Health & Sport (DHGS), 85737 Ismaning, Germany; moha.motevalli@dhgs-hochschule.de (M.M.); markus.schauer@dhgs-hochschule.de (M.S.); susanne.mair.dz@edu.dhgs-hochschule.de (S.M.)

**Keywords:** environmental microbiology, skin microbiota, *Staphylococcus aureus*, bacterial interactions, bacterial diversity, microbial persistence, hydrogen peroxide nebulization, decontamination, hygiene, microorganisms

## Abstract

Fitness centers represent high-contact environments where microorganisms can accumulate and persist on shared surfaces. This study aimed to investigate bacterial contamination dynamics on gym equipment and evaluate the effects of hydrogen peroxide (H_2_O_2_) nebulization combined with preceding professional manual cleaning on the recovery of viable surface-associated microorganisms. A two-phase investigation was conducted in a commercial fitness facility. Repeated environmental sampling was performed on 12 gym devices over five consecutive days at three daily timepoints (morning, midday, evening), followed by a pre–post assessment of the combined terminal decontamination procedure. All isolates were recovered using culture-based methods and identified by MALDI TOF MS. Species were classified by ecological origin, and persistence, diversity, and intervention effects were quantified using ecological metrics. Statistical analyses were performed in R. Across all samples, 248 isolates representing 61 species were detected, with the recovered isolates predominantly belonging to skin-associated families including Micrococcaceae (25.4%), Staphylococcaceae (24.2%), and Moraxellaceae (22.2%). Routine cleaning with an alcohol-based cleaner produced only modest reductions in recovered bacterial isolate counts (*p* = 0.173), with 68% of environmental species no longer recovered after routine cleaning, whereas 93% of skin-associated species remained detectable following routine cleaning. In contrast, the terminal decontamination procedure consisting of professional manual cleaning followed by H_2_O_2_ nebulization resulted in 88% of detected species no longer being recovered after treatment, with no Gram-negative species recovered in post-treatment cultures, while only three Gram-positive species (*Staphylococcus epidermidis*, *Staphylococcus hominis*, and *Dietzia cinnamea*) continued to be recovered after treatment. Contamination levels and persistence patterns varied markedly across devices. Gym equipment surfaces repeatedly yielded predominantly human-derived communities of recoverable viable bacteria despite routine cleaning. The combined terminal decontamination procedure produced greater reductions in recoverable bacterial species than routine overnight cleaning under the conditions evaluated, supporting further investigation of this combined intervention as a supplemental terminal decontamination strategy for high-traffic fitness environments.

## 1. Introduction

The global expansion of the fitness and wellness industry has markedly increased the use of shared exercise facilities, where large numbers of individuals interact daily with high-contact environmental surfaces [1,2]. Gym equipment handles, grips, seats, and touch interfaces are repeatedly exposed to direct skin contact, perspiration, and respiratory droplets, creating favorable conditions for microbial deposition, persistence, and transfer [3,4,5,6]. These environments may therefore act as reservoirs for transient and persistent bacterial contamination, particularly in settings characterized by high user turnover and incomplete surface decontamination [7,8,9]. Although routine cleaning is widely implemented in fitness centers, its real-world microbiological effectiveness remains poorly defined, highlighting the need to characterize equipment-specific contamination dynamics to optimize hygiene practices and better assess potential public health risks.

Environmental contamination of frequently touched surfaces has been extensively documented in healthcare and other built environments, where frequently touched environmental surfaces have been implicated in indirect microbial transmission [10,11]. Comparable mechanisms may also operate in athletic and recreational facilities, although substantially fewer studies have examined microbial ecology in fitness environments. Existing reports consistently identify the predominance of skin-associated bacteria on gym surfaces, including *Staphylococcus*, *Micrococcus*, and *Acinetobacter*, reflecting continuous microbial transfer from users [4,5,6,7,12]. These microorganisms can persist on abiotic surfaces for prolonged periods depending on environmental conditions, surface properties, and cleaning efficacy [9,11]. In this context, culture-based approaches remain particularly valuable because they quantify viable microorganisms with potential for transmission [13,14], whereas DNA-based methods may also detect non-viable residues. Matrix-assisted laser desorption/ionization time-of-flight mass spectrometry (MALDI-TOF MS) further enhances this approach by enabling high-resolution taxonomic identification of environmental isolates and detailed characterization of contamination patterns [15,16].

Routine surface cleaning in gyms typically relies on manually applied alcohol-based disinfectants or quaternary ammonium compounds [17,18,19,20]. However, the efficacy of these approaches may vary depending on application consistency, surface geometry, contact time, and microbial resilience [9,17,19,21]. Irregularly shaped handles, cable systems, and textured surfaces may hinder complete decontamination and permit residual microbial persistence [9,11]. Moreover, continuous user contact can rapidly reintroduce microorganisms, restoring microbial burden shortly after cleaning interventions [22,23]. These limitations have increased interest in no-touch automated disinfection technologies capable of achieving more homogeneous environmental coverage. Automated no-touch systems can reduce operator-dependent variability and improve the reproducibility of terminal decontamination [24]. Common approaches include hydrogen peroxide-based systems and ultraviolet-C (UV-C) irradiation; unlike UV-C, which depends on direct irradiation and may be limited by shadowing, airborne hydrogen peroxide (H_2_O_2_) can disperse throughout enclosed spaces and reach less accessible surfaces [25,26].

H_2_O_2_ vaporization and nebulization systems have emerged as effective airborne disinfection strategies due to their broad-spectrum antimicrobial activity and ability to disperse throughout enclosed environments [27,28]. Substantial reductions in environmental contamination have been demonstrated following H_2_O_2_-based terminal disinfection, particularly in healthcare settings [29,30,31,32,33]. However, evidence regarding their effectiveness in public fitness facilities remains limited, particularly with respect to their impact on the composition, persistence, and ecological distribution of surface-associated bacterial communities.

The present study aimed to characterize bacterial contamination and evaluate disinfection practices on gym equipment surfaces in a commercial fitness facility using repeated environmental sampling and culture-based microbial identification. In contrast to predominantly descriptive studies relying on narrow, single-time-point sampling designs [5,12,34,35], this investigation integrated repeated-measures sampling with ecological classification of isolates, temporal persistence analysis, and evaluation of H_2_O_2_ nebulization combined with preceding professional manual cleaning. Additionally, the study examined contamination patterns across multiple device types and quantified the effectiveness of these two disinfection approaches in reducing viable bacterial contamination. By integrating repeated-measures sampling, ecological classification of isolates, persistence analysis, and assessment of combined terminal decontamination procedure, this study extends beyond descriptive contamination surveys by providing insights into bacterial persistence patterns and the effectiveness of different hygiene interventions in a real-world fitness environment. The ultimate objective was to generate evidence to support targeted hygiene interventions that minimize microbial exposure risks in fitness facilities.

## 2. Materials and Methods

### 2.1. Study Design

The present two-phase study employed a repeated-measures sampling design combined with a pre-post intervention assessment. All sampling took place in a fitness studio located in Munich, Germany. The repeated-measures sampling was carried out over five consecutive weekdays (Monday to Friday) to characterize temporal variation in environmental microbial contamination associated with routine facility use (Phase 1). In addition, a separate pre-post intervention sampling was performed on a Tuesday evening to evaluate a terminal decontamination procedure consisting of professional manual cleaning followed by H_2_O_2_ nebulization under real-world operating conditions (Phase 2). Together, these complementary phases were designed to assess the efficacy of routine and enhanced disinfection practices in reducing contamination linked to human activity and environmental exposure.

### 2.2. Study Protocol and Sampling Design

A set of 12 gym devices encompassing a broad range of equipment types, including resistance-based, cable-operated, and cardiovascular machines, was systematically selected for microbiological assessment. The analysis included twelve gym devices: (1) functional trainer (wire rope hoist), (2) lat pulldown (wire rope hoist), (3) Roman chair, (4) pulldown and seated row combo, (5) ab crunch machine, (6) leg extension, (7) reverse pec deck (butterfly reverse), (8) 45° leg press, (9) arm ergometer (wheel 1), (10) seated row machine (wheel 2), (11) upper body ergometer, and (12) treadmill. Devices were selected based on differences in user interaction, surface material, and handle design, affecting microbial transfer and persistence. Samples were taken from device handles as the primary hand-contact surfaces and key sites for microbial exposure, with a predefined handle region consistently targeted across all sampling events. Approximately 25 cm^2^ (5 × 5 cm) of the handle surface was sampled for each collection using a standardized sampling area. For repeated sampling, adjacent standardized areas within the predefined handle region were sampled to minimize the potential influence of previous swabbing on subsequent bacterial recovery while maintaining comparable surface characteristics.

Repeated measures sampling (Phase 1) was performed three times per day at fixed time points: morning before opening, midday at 12:00, and evening prior to closing. Morning samples were collected after routine overnight cleaning and therefore reflected a post-disinfection baseline. Midday samples captured peak-day microbial build-up, whereas evening samples indicated total contamination following prolonged use. A total of 180 environmental samples were collected, representing 15 samples per device over the five-day study period within the structured sampling design.

To evaluate the efficacy of hydrogen peroxide (H_2_O_2_) nebulization, an additional two-stage environmental sampling protocol (Phase 2) was implemented under real-world operating conditions. Environmental sampling was performed during two consecutive intervention phases. The first sampling campaign was conducted on a Tuesday evening immediately after the end of public operating hours and on a separate day from the repeated-measures sampling period. The second sampling campaign was subsequently conducted following cleaning and terminal disinfection performed by a professional hygiene service provider. A total of 24 environmental samples (12 devices sampled before and 12 devices sampled after the intervention) were collected during Phase 2.

### 2.3. Environmental Sample Collection and Processing

Sterile ESwab^TM^ collection systems with liquid Amies transport medium were used for environmental sampling. Sampled surface area was standardized across all devices and time points to ensure comparability of measurements. A standardized multidirectional swabbing technique with consistent pressure and coverage was used to minimize operator-related variability in microbial recovery. To increase microbial recovery, the swab tip was moistened with transport medium before sampling. All samples were transported to the laboratory immediately after collection and processed within a standardized time frame to maintain bacterial viability. Swabs were stored in transport medium at ambient temperature until processing to minimize changes in microbial composition.

### 2.4. Cleaning and Disinfection Protocol

Routine pre-cleaning was carried out using the alcohol-based cleaner NEOfris classic (Dreiturm, Steinau an der Straße, Germany) according to the manufacturer’s instructions (50–100 mL diluted in 8 L water). Airborne disinfection intervention was performed using the NOCOSPRAY^®^ nebulization system (OXY’PHARM, Champigny-sur-Marne, France), which disperses a stabilized 12% H_2_O_2_ solution as a dry mist. The nebulization system was operated according to the manufacturer’s validated application protocol appropriate for the treated room volume. Depending on room size, the system is designed for spaces ranging from 10 to 250 m^3^, 20 to 500 m^3^, or 40 to 1000 m^3^. The device operates via an electric turbine system (1100 W; 22,000 rpm) and generates nebulization through a Venturi-effect mechanism without the use of a liquid injection pump. The aerosolized disinfectant is distributed up to 15 m from the device with a mist exit velocity of approximately 80 m/s, enabling homogeneous spatial distribution within the treated environment. The nebulization procedure was performed in accordance with the manufacturer’s recommendations for the treated room volume and application dosage. During the disinfection cycle, the treated area remained closed to ensure sufficient exposure and contact time of the aerosolized H_2_O_2_. Following completion of the manufacturer-recommended exposure period, the treated area was ventilated and aerated prior to re-entry. Post-treatment environmental samples were collected after completion of the aeration phase.

The Phase 2 intervention consisted of a single terminal decontamination procedure comprising professional manual surface cleaning immediately followed by H_2_O_2_ nebulization. No environmental sampling was performed between these two steps. Consequently, the study was designed to evaluate the effectiveness of the overall terminal decontamination procedure rather than the independent contribution of manual cleaning and H_2_O_2_ nebulization. Phase 2 was exploratory and focused on the immediate post-treatment effect; repeated application and long-term effectiveness or safety were not evaluated.

### 2.5. Culture-Based Isolation Procedures

All workflow steps were conducted in accordance with recognized microbiological standards and widely applied guidelines for culture-based isolation and characterization [36]. A 10 µL aliquot from each ESwab transport medium was inoculated onto several culture media to achieve broad bacterial recovery and targeted isolation of clinically important groups. The culture panel comprised Müller–Hinton agar with 5% sheep blood for general cultivation, mannitol salt agar for staphylococcal selection, and Endo agar for identifying Gram-negative species. To capture microbial heterogeneity, colonies were selected based on distinct morphological traits, including differences in size, color, shape, and texture. When multiple morphotypes were present on a plate, colonies representing each type were picked to ensure within-sample diversity.

Because representative colonies were selected based on distinct colony morphology for MALDI-TOF MS identification, the isolation workflow was designed to characterize the diversity and occurrence of cultivable bacteria rather than to quantify microbial burden. Although quantitative colony counts were recorded for selected cultures, systematic colony forming unit (CFU) enumeration was not performed across all environmental samples. Accordingly, the number of recovered isolates corresponds to the representative colonies chosen for identification and does not reflect CFUs, bacterial load, or any measure of quantitative bacterial abundance. Recovered isolate counts should therefore not be interpreted as the number of positive environmental samples, because a single environmental sample could yield multiple morphologically distinct bacterial colonies representing different recovered isolates.

Following inoculation, plates were incubated aerobically at 37 °C and evaluated after approximately 24 h. Plates showing low or delayed growth were re-examined after extended incubation to detect slow-growing species. After incubation, morphologically distinct colonies were transferred to fresh media to obtain pure cultures prior to identification. Sterility of all culture media was verified before use, and all sample handling followed strict aseptic procedures. Negative controls, consisting of unused swabs exposed to the sampling environment and processed identically to study samples, were included periodically throughout the study. No bacterial growth was observed in any negative-control culture, indicating the absence of detectable environmental or laboratory contamination during sample collection and processing. Standard microbiological quality control measures were applied throughout the study to safeguard data integrity and prevent external contamination.

### 2.6. Bacterial Identification and Analytical Classification

Bacterial identification was performed using MALDI-TOF MS (VITEK^®^ MS system; bioMérieux, Lyon, France) using α-cyano-4-hydroxycinnamic acid (CHCA) matrix, with the commercial Knowledge Base v2.0 database for clinical use. Identification results were interpreted according to the manufacturer’s recommended confidence criteria for species- and genus-level assignments. Instrument calibration and routine quality-control procedures were performed according to the manufacturer’s instructions prior to analysis. Bacterial contamination of gym equipment was defined as the detection and successful identification of at least one viable isolate following culture. Accordingly, all subsequent analyses were based on recovered isolates and species detections. Relative frequencies presented throughout the manuscript refer to the proportion of recovered isolates assigned to each taxonomic group rather than quantitative bacterial abundance on sampled surfaces. Spectral profiles were compared with a validated reference database, and identifications were interpreted using manufacturer-specified score criteria. Scores meeting validated cut-off values supported species-level assignment, whereas lower scores were reported at the genus level. Presumptive *Staphylococcus aureus* isolates identified by MALDI-TOF MS were additionally confirmed phenotypically using a commercial coagulase latex agglutination assay (Pastorex^TM^ Staph-Plus, Bio-Rad Laboratories, Marnes-la-Coquette, France), performed according to the manufacturer’s instructions. Confirmed isolates were stored at −80 °C in cryoprotective cryobank tubes to ensure long-term preservation and availability for future molecular or phenotypic analyses.

Each isolate was classified according to its ecological origin as either skin-associated or environmental based on established descriptions of microbial habitats and taxon-specific ecological characteristics [37,38,39,40,41]. Species commonly regarded as members of the normal human skin microbiota were classified as skin-associated, whereas species predominantly isolated from soil, water, or other environmental reservoirs were classified as environment-associated. Species occupying multiple ecological niches were classified according to their most frequently reported primary habitat. Species richness and family richness for each device were defined as the number of unique species or families detected across all sampling events. Temporal persistence was defined as the proportion of sequential sampling intervals for a given device in which at least one bacterial species detected during one sampling interval was also recovered during the immediately subsequent sampling interval. Persistence (%) was calculated as: (Number of consecutive sampling intervals showing repeated recovery of at least one bacterial species ÷ Total number of consecutive sampling intervals evaluated for that device) × 100. Persistence therefore reflects repeated species recovery at the device level and was not calculated for individual bacterial strains. Taxonomic ranks were assigned according to MALDI-TOF MS database classifications, and diversity metrics were derived directly from observed detections without rarefaction or additional normalization.

### 2.7. Statistical Analysis

All statistical analyses were carried out using R (version 4.5.1). Data processing and visualization were conducted using standard ecological and statistical workflows for microbial community data. Taxonomic composition across devices was visualized with heatmaps displaying the relative frequency of recovered isolates, without accompanying inferential tests. Temporal trends over the five-day sampling period were assessed using a simple linear regression model with day (1–5) as a continuous predictor, with significance evaluated via a two-sided test of the null hypothesis that the slope equals zero (i.e., no linear change in the outcome across days). The effect of routine overnight cleaning on recovered bacterial isolate counts was evaluated using paired evening and subsequent-morning observations from the same devices. Four consecutive overnight intervals across 12 devices yielded 48 device-level evening–morning pairs. Recovered isolate counts were log-transformed [log_10_(total recovered isolates + 0.5)], and normality of the within-pair differences was assessed using the Shapiro-Wilk test. Because the paired differences showed a significant departure from normality (W = 0.904, *p* < 0.001), evening and subsequent-morning observations were compared using the Wilcoxon signed-rank test. Species-level differences between evening and morning sampling periods were examined using Fisher’s exact test based on aggregated species detection frequencies across sampling events rather than matched binary outcomes for individual device-level pairs. These species-level analyses were considered exploratory, and no formal adjustment for multiple comparisons was applied. Species were subsequently classified as no longer recovered after cleaning, reduced detection frequency, or repeatedly recovered-based on post-cleaning culture results. The efficacy of the terminal decontamination procedure was assessed descriptively; the proportion of species no longer recovered after treatment was calculated, and results were stratified by Gram-staining category. A two-sided *p*-value < 0.05 was considered statistically significant for all tests.

## 3. Results

A total of 248 bacterial isolates were recovered from 180 samples, representing 32 families and 61 species. Among the recovered isolates, 71.8% belonged to three families: Micrococcaceae (63 isolates, 25.4%), Staphylococcaceae (60 isolates, 24.2%), and Moraxellaceae (55 isolates, 22.2%), all of which are commonly associated with the human skin microbiota. *Micrococcus luteus* (n = 60) was the most frequently recovered species among the identified isolates, followed by *Acinetobacter lwoffii* (n = 47) and *Staphylococcus saprophyticus* (n = 17). Several clinically relevant species were also identified, including *Staphylococcus aureus* (n = 6), *Pseudomonas stutzeri* (n = 2), *Enterococcus faecalis* (n = 2), and *Stenotrophomonas maltophilia* (n = 4), although these occurred at substantially lower frequencies. A detailed breakdown of the distribution of bacterial families is provided in Table S1 of the Supplementary Materials.

Across the three daily sampling periods, the number of recovered bacterial isolates varied descriptively over the course of the day. Morning samples collected after routine overnight cleaning yielded 68 isolates (27.4% of all recovered isolates), compared with 91 isolates (36.7%) at midday and 89 isolates (35.9%) in the evening. Thus, isolate recovery was lowest in the morning, whereas midday and evening samples yielded higher and broadly comparable numbers of recovered isolates.

**Figure 1:**
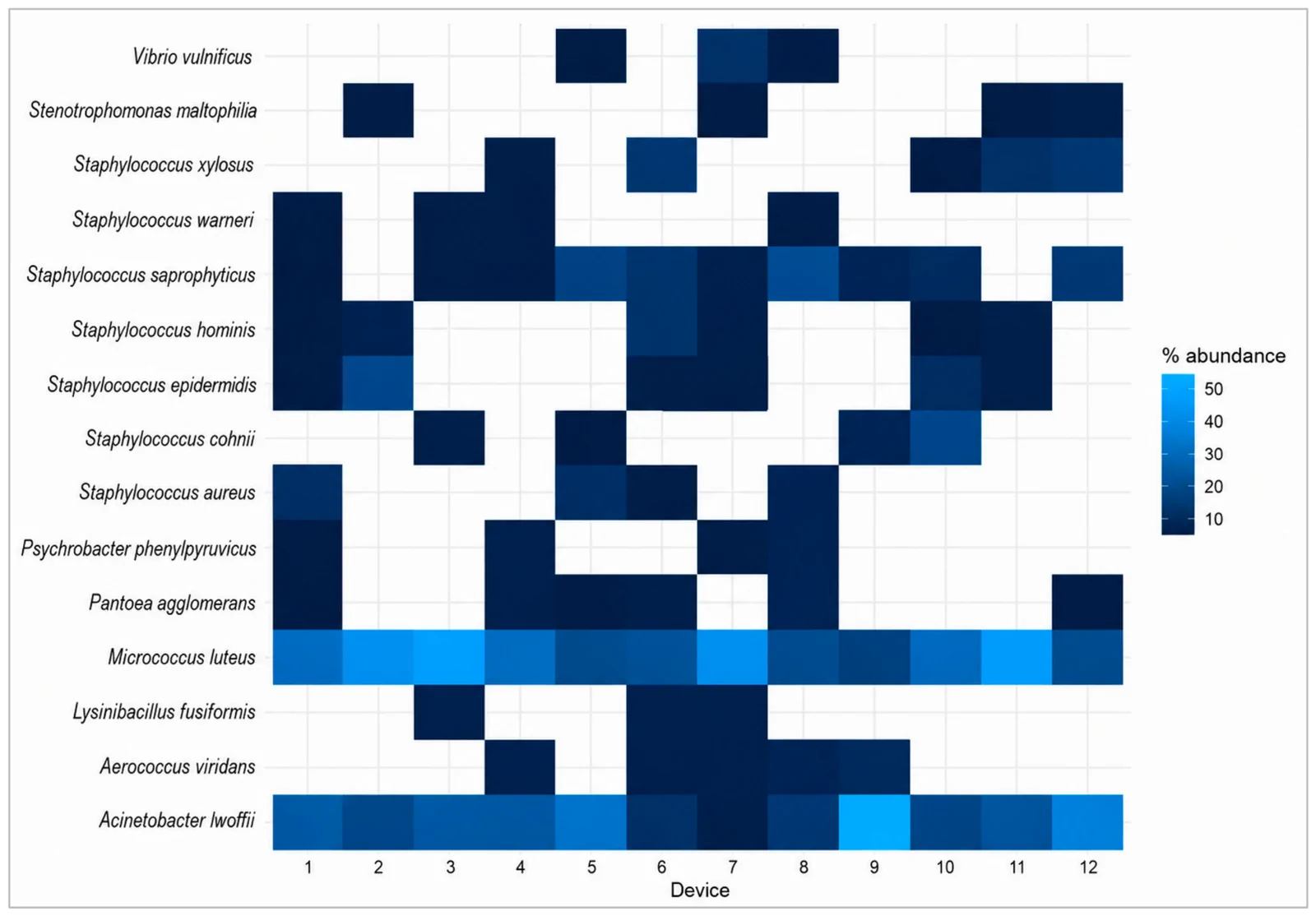
The 15 most frequently identified bacterial species across 12 gym equipment surfaces. Data are shown as a heatmap, where color intensity reflects the relative frequency (%) of recovered isolates and lighter shades indicate higher frequency. Gym devices: (1) functional trainer, (2) lat pulldown, (3) Roman chair, (4) pulldown combo, (5) ab crunch machine, (6) leg extension, (7) reverse pec deck, (8) 45° leg press, (9) seated row machine, (10) arm ergometer, (11) upper body ergometer, (12) treadmill.

**Figure 2:**
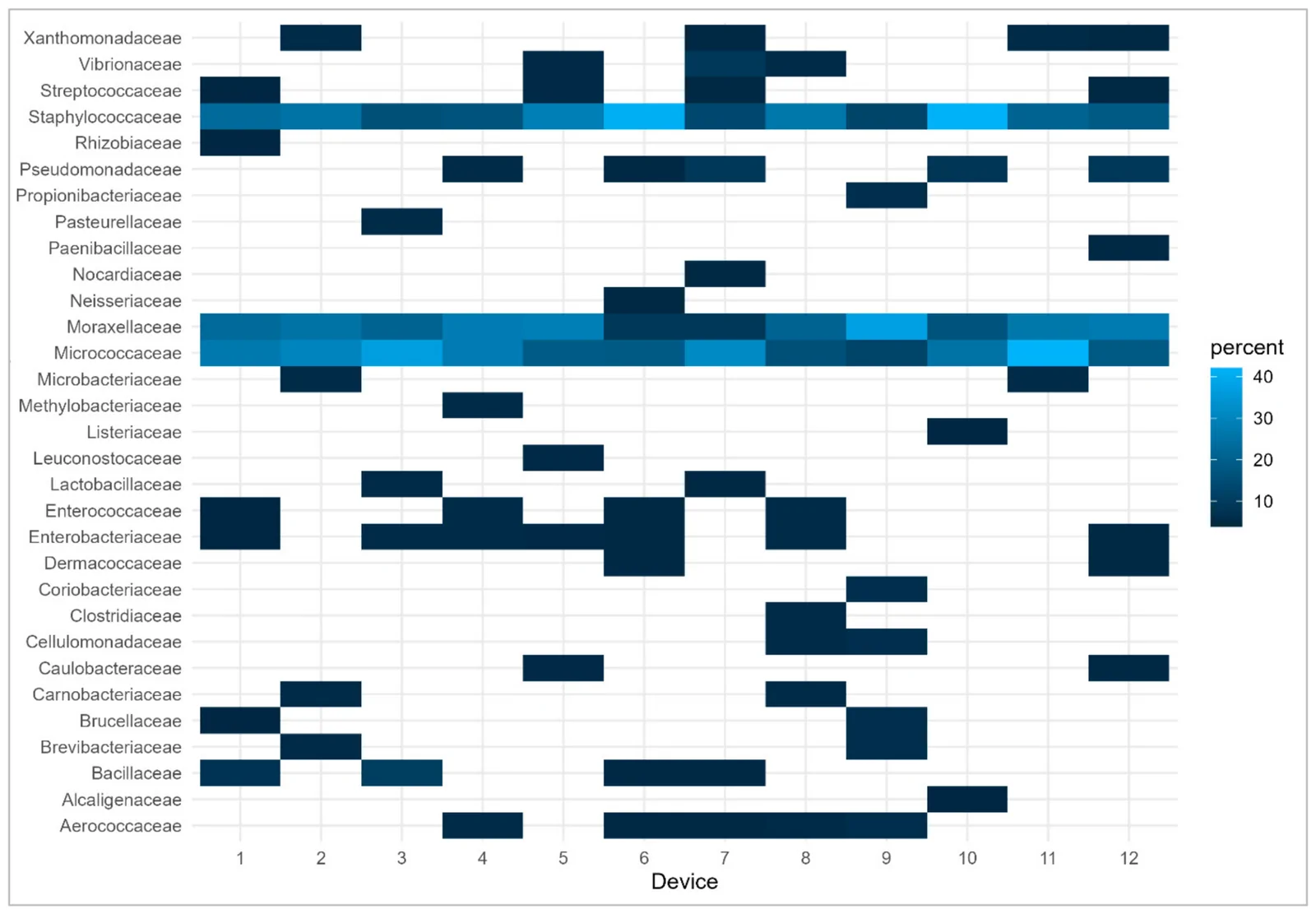
Bacterial family composition across 12 gym equipment surfaces. Data are shown as a heatmap, with color intensity reflecting the relative frequency (%) of recovered isolates and lighter shades indicating higher frequency. Gym devices: (1) functional trainer, (2) lat pulldown, (3) Roman chair, (4) pulldown combo, (5) Ab crunch machine, (6) leg extension, (7) reverse pec deck, (8) 45° leg press, (9) seated row machine, (10) arm ergometer, (11) upper body ergometer, (12) treadmill.

The highest number of recovered bacterial isolates across all sampled gym devices was observed on the functional trainer (26 isolates; 11% of all recovered isolates), followed by the arm ergometer (24 isolates; 10%), while the seated row machine demonstrated the lowest number of detections (16 isolates; 6%). Overall, recovered bacterial isolates were broadly distributed among the sampled devices, with only moderate variation in contamination frequency. Across the 61 distinct microbial species identified, the functional trainer harbored the greatest species diversity (16 species; 26%), whereas the upper body ergometer showed the lowest diversity (9 species; 15%). Across the 32 microbial families detected, the reverse pec deck demonstrated the highest family-level diversity (11 families; 34%), while the lower family-level diversity was observed on the upper body ergometer (5 families; 16%). Figure 1 presents a heatmap demonstrating the distribution patterns of the top 15 bacterial species identified across the sampled gym devices, while Figure 2 shows the distribution patterns of bacterial families detected across the gym devices.

Across all devices, the arm ergometer showed the highest proportion of recovered *Staphylococcus* isolates (10 detections; 42% of all recovered isolates from that device), whereas the seated row exhibited the lowest proportion (2 detections; 12%). Skin-associated isolates predominated across all gym devices. The upper body ergometer demonstrated the highest proportion of skin-associated taxa (17 detections; 89% of all recovered isolates from that device), followed by the arm ergometer (20; 83%) and lat pulldown (16; 80%). Even the lowest proportion, observed on the reverse pec deck (12; 54%), indicated that more than half of the recovered isolates from that device were skin-associated. Environmental isolates accounted for a smaller proportion of recovered isolates overall, although their relative frequency varied by device. The reverse pec deck exhibited the highest proportion of environmental taxa (10 detections; 46% of all recovered isolates from that device), whereas the upper body ergometer showed the lowest proportion (2; 11%).

Persistence of microbial species across consecutive samplings differed notably among devices. The ab crunch machine demonstrated the highest persistence rate (55.6%), whereas the lat pulldown exhibited the lowest persistence rate (22.2%). Two gym devices showed significant day-to-day increases in isolate recovery frequency across the five-day sampling period: the ab crunch machine (*p* = 0.012) and upper body ergometer (*p* = 0.006). The reverse pec deck displayed a non-significant upward trend (*p* = 0.07), while all remaining devices showed stable detection frequencies across the sampling days (*p* > 0.05). Table 1 presents the device-specific isolate recovery frequencies, diversity metrics, contamination sources, and temporal patterns. Device-specific evening–morning isolate recovery patterns are summarized in Table S2 of the Supplementary Materials.

**Figure 3:**
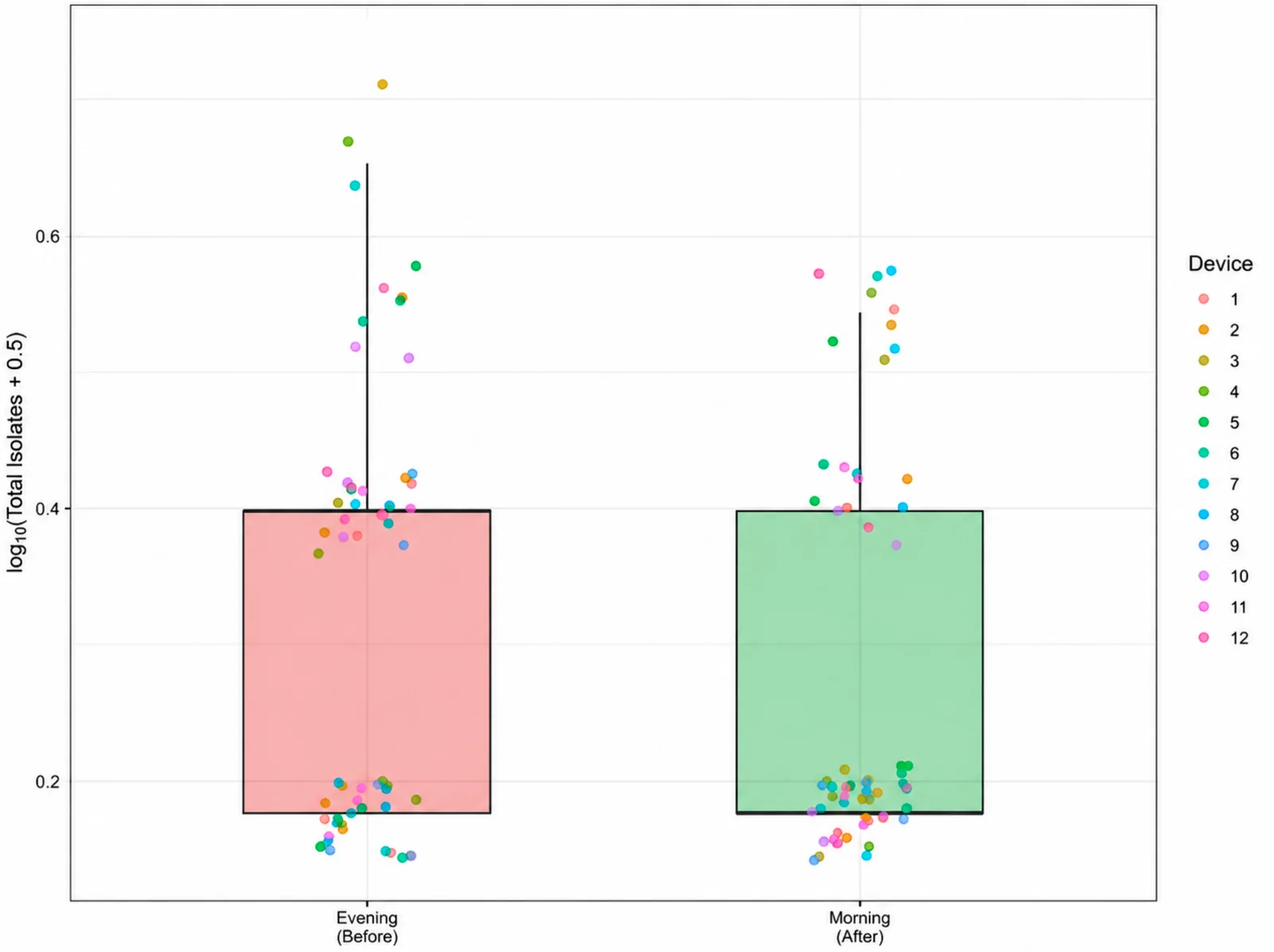
Effect of routine cleaning on recovered bacterial isolate counts. Boxplots compare log-transformed recovered bacterial isolate counts (log_10_(recovered isolates + 0.5)) for evening and corresponding subsequent-morning samples. The horizontal line within each box represents the median, the lower and upper box boundaries represent the first and third quartiles, respectively, and whiskers extend to values within 1.5× the interquartile range. Jittered points represent individual device-level observations and were jittered slightly in the graphical display to reduce overlap. The underlying transformed values were calculated directly from integer recovered-isolate counts (e.g., n = 0, −0.301; n = 1, 0.176; n = 2, 0.398; n = 3, 0.544). The figure includes 48 paired device-level observations across four consecutive overnight intervals and 12 devices. Differences were evaluated using the Wilcoxon signed-rank test (*p* = 0.173).

Routine overnight cleaning was associated with a modest reduction in recovered bacterial isolate counts. The median recovered isolate count was 1 in both evening and subsequent-morning samples, while the corresponding means were 1.71 and 1.48 isolates, respectively. The paired difference was not statistically significant (Wilcoxon signed-rank test, *p* = 0.173; rank-biserial r = 0.18). Individual device-level observations showed variability in recovered isolate counts in both evening and subsequent-morning samples, with considerable overlap between the two distributions, although some observations showed marked differences approaching near-zero morning values. The efficacy of routine overnight cleaning in reducing total recoverable bacterial isolates on gym equipment surfaces is summarized in Figure 3.

**Figure 4:**
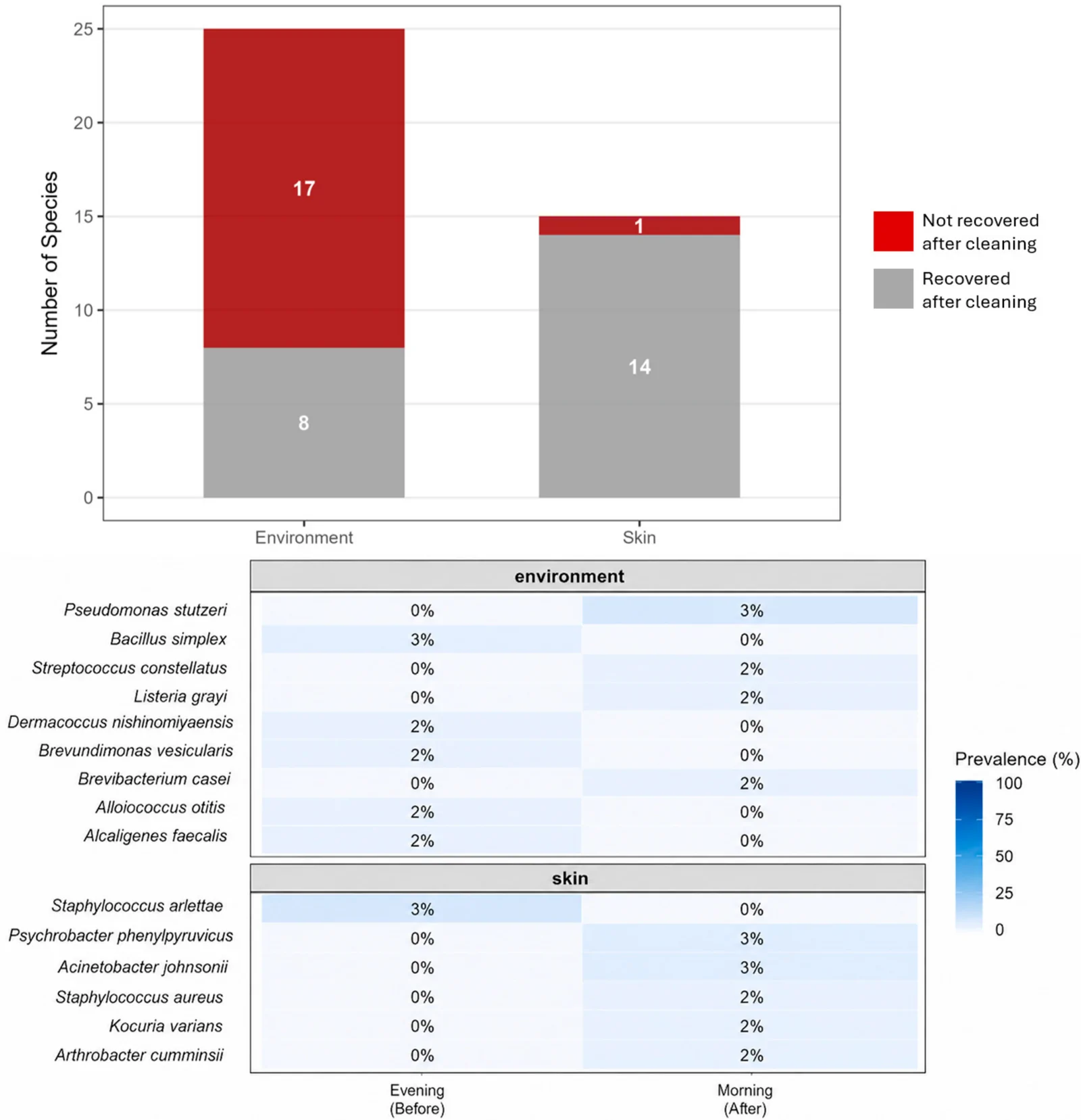
Recovery of environmental bacteria following routine cleaning. (**Upper panel**): Comparison of the post-cleaning recovery status of bacterial species categorized by ecological niche. Red indicates species that were no longer recovered after cleaning; grey indicates species that continued to be recovered after cleaning. Environmental species showed a higher proportion of species no longer recovered after cleaning (17/25) compared with skin-associated species (1/15). (**Lower panel**): Prevalence heatmap for top differential species (Evening vs. Morning). Color intensity corresponds to the percentage of samples in which the species was detected. Species-level detection frequencies were aggregated across four consecutive overnight intervals and 12 devices (48 device-level evening–morning comparisons) and analyzed using Fisher’s exact test.

To evaluate the efficacy of routine cleaning on surface microbial communities, we analyzed the differential detection frequency and prevalence of 40 bacterial species categorized by their primary niche: environment-associated or skin-associated flora. Exploratory comparison of evening and morning detection frequencies indicated variation among selected taxa. A total of 18 species were no longer recovered following the cleaning procedure. The greatest reduction in recovery was observed among environmental species, with several species no longer detected in post-cleaning cultures. In contrast, skin-associated species exhibited higher resilience, remaining detectable more frequently after cleaning. Categorical analysis of the species’ outcomes further highlighted the differential effect of routine cleaning. Of the 25 environmental species detected before cleaning, 17 (68%) were no longer recovered after cleaning, whereas 8 continued to be recovered. Conversely, the skin-associated community was largely preserved; only 1 out of 15 skin-associated species (~7%) was no longer recovered, while the remaining 14 species continued to be recovered following cleaning (Figure 4, upper panel). Prevalence heatmaps of the top differential species support this pattern, showing a clear reduction in taxa such as *Bacillus simplex* and *Staphylococcus arlettae* after cleaning, whereas species like *Pseudomonas stutzeri* and *Psychrobacter phenylpyruvicus* exhibited higher detection frequencies in the morning samples (Figure 4, lower panel).

**Figure 5:**
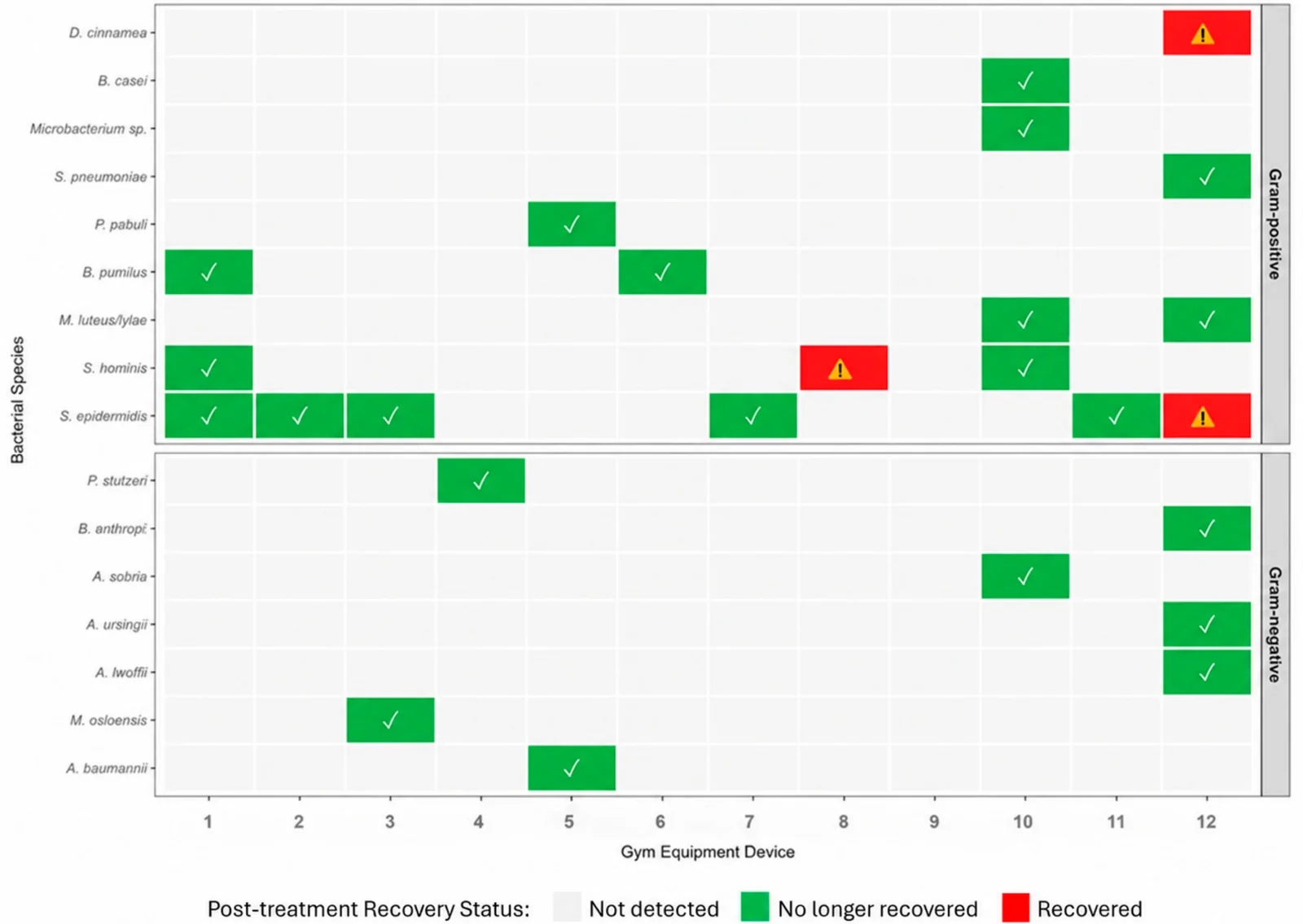
Effectiveness of the terminal decontamination procedure (manual cleaning followed by H_2_O_2_ nebulization) against bacterial species recovered from gym equipment. A total of 25 bacterial species initially detected on the devices were evaluated. Following completion of the terminal decontamination procedure, 22 species were no longer recovered after treatment (green), whereas 3 species (*Dietzia cinnamea*, *Staphylococcus hominis*, and *Staphylococcus epidermidis*) continued to be recovered after treatment (red). Grey indicates species that were not detected on the corresponding device either before or after treatment. Gym devices: (1) functional trainer, (2) lat pulldown, (3) Roman chair, (4) pulldown combo, (5) ab crunch machine, (6) leg extension, (7) reverse pec deck, (8) 45° leg press, (9) seated row machine, (10) arm ergometer, (11) upper body ergometer, (12) treadmill.

To evaluate the effectiveness of the terminal decontamination procedure consisting of professional manual cleaning followed by H_2_O_2_ nebulization, 25 bacterial species identified on gym equipment were tracked pre- and post-treatment. The terminal decontamination procedure demonstrated high overall effectiveness, with 88% of the detected species no longer recovered following treatment. A descriptive pattern was observed according to Gram-staining category. No Gram-negative species (including *Pseudomonas stutzeri*, *Brucella anthropi*, *Aeromonas sobria*, *Acinetobacter ursingii*, *Acinetobacter lwoffii*, *Moraxella osloensis*, and *Acinetobacter baumannii*) were recovered in post-treatment cultures from any of the tested devices. In contrast, continued recovery after treatment was exclusively observed among Gram-positive taxa. Specifically, *Staphylococcus epidermidis*, *Staphylococcus hominis*, and *Dietzia cinnamea* remained detectable on Device 8 (45° leg press) and Device 12 (treadmill) following the nebulization intervention. However, the remaining Gram-positive species including *Bacillus pumilus*, *Paenibacillus pabuli*, *Streptococcus pneumoniae*, *Microbacterium*, *Micrococcus luteus/lylae*, and *Brevibacterium casei* were no longer recovered following treatment (Figure 5).

## 4. Discussion

This study provides a real-world evaluation of hydrogen peroxide (H_2_O_2_) nebulization combined with preceding professional manual cleaning on gym equipment surfaces using environmental sampling and MALDI-TOF MS identification. Four principal findings emerged. First, gym equipment surfaces harbored a diverse bacterial community dominated by skin-associated taxa among recovered isolates (72% of isolates), particularly *Micrococcus luteus*, *Acinetobacter lwoffii*, and several *staphylococcal* species including *S. epidermidis*, *S. hominis*, *S. xylosus*, *S. cohnii*, *S. aureus*, *S. warneri*, *S. arlettae*, and *S. saprophyticus*. Second, routine overnight cleaning was associated with a modest, non-significant reduction in recovered bacterial isolate counts (*p* = 0.173) and showed selective efficacy, with 68% of environmental species no longer recovered after cleaning, whereas 93% of skin-associated species continued to be recovered following cleaning. Third, the terminal decontamination procedure consisting of professional manual cleaning followed by H_2_O_2_ nebulization demonstrated substantially greater efficacy, with 88% of detected species no longer recovered after treatment, including no Gram-negative species recovered in post-treatment cultures, whereas three Gram-positive species (*Staphylococcus epidermidis*, *Staphylococcus hominis*, and *Dietzia cinnamea*) continued to be recovered after treatment on selected devices. Fourth, contamination patterns and disinfection efficacy varied considerably between devices, with some equipment such as the 45° leg press and treadmill demonstrating continued recovery of bacterial species following both interventions.

The predominance of skin-associated taxa indicates that gym equipment microbiota are primarily shaped by continuous human shedding and direct-contact transfer. More than 70% of isolates belonged to Micrococcaceae, Staphylococcaceae, and Moraxellaceae, consistent with built-environment microbiome theory describing progressive “humanization” of indoor microbial ecosystems through repeated occupancy and surface interaction [42,43,44]. However, this process varied across devices according to contact intensity and usage patterns. Ergometers showed particularly high proportions of skin-associated microorganisms, suggesting selective enrichment of bacteria adapted to survival on skin and dry abiotic surfaces. This observation is biologically plausible given the marked desiccation tolerance of *Micrococcus* and coagulase-negative *Staphylococcus* species [11,45,46]. Similarly, the repeated recovery of *Acinetobacter lwoffii* across consecutive sampling intervals suggests that this species was consistently associated with frequently touched gym surfaces. Nevertheless, because strain-level molecular typing was beyond the scope of the present study, these repeated recoveries cannot distinguish between long-term persistence of the same bacterial strain and repeated reintroduction of genetically distinct strains belonging to the same species through continued human contact.

Although most recovered isolates likely represented low-pathogenicity commensals, the recovery of species with recognized opportunistic pathogenic potential, including *Staphylococcus aureus*, *Enterococcus faecalis*, *Stenotrophomonas maltophilia*, and *Pseudomonas stutzeri*, is relevant to environmental hygiene surveillance. However, their recovery from surfaces does not demonstrate transmission or infection risk. Prior investigations similarly reported contamination of fitness equipment with pathogenic and multidrug-resistant *staphylococci* [6,12,47]. Notably, some studies documented widespread *S. aureus* contamination in fitness facilities [6,47], whereas others identified elevated antimicrobial resistance gene abundance on gym surfaces [12]. However, the overall contamination profile appears more indicative of continuous commensal microbial exchange than extensive pathogenic colonization, as clinically meaningful transmission additionally depends on inoculum size, virulence, host susceptibility, and environmental persistence [11,22,48,49].

Several devices demonstrated increasing contamination across the five-day sampling period, while persistence rates remained substantial across nearly all equipment categories, supporting the concept of continuous equilibrium between cleaning-mediated removal and human-mediated re-seeding [50]. Higher persistence on selected devices likely reflects the environmental resilience of skin-associated bacteria together with repeated deposition through frequent user contact [51,52,53,54]. Particularly strong accumulation trends on the ab crunch machine and upper body ergometer suggest that repetitive gripping and prolonged contact facilitate deposition of skin-associated microorganisms, perspiration residues, and organic debris that enhance microbial persistence. Differences in the duration and intensity of hand contact may therefore contribute to device-specific recovery patterns; however, actual contact duration, grip intensity, and device-specific usage frequency were not measured, and these interpretations should be considered hypothesis-generating. Similar accumulation dynamics have been described in healthcare facilities, transportation systems, and other high-occupancy built environments [55,56,57].

Although contamination was widespread across all devices, substantial differences emerged in species richness, persistence rates, and staphylococcal prevalence. Elevated contamination on cable-operated and hand-intensive devices likely reflects repeated microbial transfer from hands together with microbial retention on textured surfaces and nutrient deposition from perspiration and skin lipids [58,59]. Similar patterns have been reported on high-contact fitness equipment [6]. Collectively, these findings indicate that patterns of recoverable bacterial contamination are associated more closely with contact intensity and persistence dynamics rather than equipment category alone. Consequently, targeted prioritization of high-contact, high-persistence equipment may provide more effective reduction in recovered bacterial isolates than uniform cleaning strategies in large commercial fitness facilities.

A central finding of the present study is that routine overnight cleaning reduced recoverable bacterial isolates only modestly and failed to achieve universal decontamination. Although overall isolate counts declined after cleaning, substantial residual contamination persisted across many devices, consistent with evidence that routine cleaning exerts selective rather than comprehensive antimicrobial effects [60,61]. Environmental species showed a greater reduction in recovery following cleaning than skin-associated microorganisms, a descriptive pattern consistent with more frequent continued recovery of human-associated bacteria after routine disinfection. This resilience is likely attributable to physiological traits including desiccation tolerance, oxidative stress resistance, and biofilm formation, together with practical factors such as incomplete surface coverage, organic load, and complex surface topography that may reduce disinfectant performance [32,45,62,63,64,65]. Unlike ATP-based assessments, which provide an indirect measure of surface cleanliness without specifically quantifying viable microorganisms [18,66], the present culture-based findings demonstrate persistence of viable skin-associated bacteria despite routine cleaning. Collectively, these results suggest that overnight cleaning alone may be insufficient to maintain low numbers of recoverable viable bacteria in heavily utilized fitness facilities because continuous user-mediated recolonization likely restores contamination rapidly following reopening. Alternative delivery approaches, such as spray-based application of conventional disinfectants, may also improve surface coverage compared with manual cleaning; however, their effectiveness remains dependent on disinfectant properties, application parameters, contact time, and accessibility of contaminated surfaces [67,68].

The terminal decontamination procedure comprising manual cleaning followed by H_2_O_2_ nebulization resulted in 22 of the 25 tracked species no longer being recovered after treatment and appeared more effective than routine overnight cleaning. These findings are consistent with previous evidence demonstrating broad-spectrum antimicrobial efficacy of hydrogen peroxide-based environmental disinfection systems [27,28,29,30,31,32,33], although, to our knowledge, no previous study has specifically evaluated the combined effectiveness of manual cleaning followed by H_2_O_2_ nebulization in public indoor environments. One study reported a 96.5% reduction in hospital environmental microbial burden following dry hydrogen peroxide implementation [30]. Similarly, healthcare studies repeatedly demonstrate that vaporized or aerosolized H_2_O_2_ technologies achieve more homogeneous environmental coverage than manual surface wiping alone [29,31,32]. The absence of Gram-negative species in post-treatment cultures was a notable descriptive observation. This pattern may be considered in the context of evidence that the Gram-negative bacterial envelope undergoes homeostatic and adaptive responses to oxidative stress [69]; however, the present study did not investigate the underlying mechanisms or formally assess an association between cell wall structure and post-treatment recovery. The continued recovery of a small number of Gram-positive species suggests potential differences in susceptibility to the terminal decontamination procedure.

Although H_2_O_2_ nebulization demonstrated high antimicrobial efficacy, its implementation requires consideration of operator and occupant safety, material compatibility, and potential effects on sensitive indoor materials and plants. Hydrogen peroxide generates reactive oxygen species, including hydroxyl radicals, which inactivate microorganisms through oxidative damage to cellular components [70,71]. However, these oxidative properties require appropriate safety measures, as concentrated H_2_O_2_ exposure may pose risks to operators and contribute to material degradation with repeated or improper use [72]. Therefore, application should be conducted under controlled conditions with validated concentrations, exposure times, restricted access during treatment, and appropriate protection or removal of sensitive items and plants from the treatment area. When properly implemented, the substantial reduction in viable microorganisms may outweigh these risks, particularly in high-traffic environments where routine cleaning alone provides insufficient microbial control.

Overall, the present study is among the first to evaluate routine overnight cleaning and a combined terminal decontamination procedure involving H_2_O_2_ nebulization preceding professional manual cleaning in a real-world gym environment using repeated culture-based assessment. The findings further support the concept that fitness facilities are dynamic built-environment microbial ecosystems shaped by human occupancy and contact behavior. Importantly, the culture-based design enabled characterization of viable microorganisms with realistic exposure potential, overcoming limitations of sequencing-based approaches that may overestimate biologically relevant contamination through detection of residual DNA. Although contamination was dominated by skin-associated commensals, persistence of viable opportunistic microorganisms following cleaning supports continued hygiene optimization and layered infection-prevention strategies incorporating routine cleaning, hand hygiene, targeted disinfection, and periodically enhanced decontamination. It is noteworthy that fungal microorganisms represent an additional component of the microbiota of fitness environments; however, their presence and response to H_2_O_2_-based disinfection were beyond the scope of the present study, which focused exclusively on bacterial contamination.

Several limitations should nevertheless be acknowledged. First, sampling was conducted within a single commercial fitness facility, which may limit external generalizability across facilities with different occupancy patterns, ventilation systems, environmental conditions, or hygiene protocols. Second, culture-based approaches inherently may underestimate total microbial diversity because non-culturable microorganisms remain undetected [73]. Third, antimicrobial resistance profiling and genomic characterization were not performed, preventing assessment of resistance determinants or strain-level transmission dynamics. Moreover, although quantitative colony counts were recorded for selected cultures, systematic CFU enumeration was not performed across all environmental samples, as representative colonies were selected based on distinct colony morphology for MALDI-TOF MS identification. Accordingly, the study was designed primarily to characterize cultivable bacterial diversity and detection patterns rather than quantitative bacterial burden, and the reported numbers of recovered isolates should not be interpreted as CFUs or absolute bacterial abundance on sampled surfaces. Finally, user-related factors such as hand hygiene, sweating intensity, and duration of equipment use, as well as environmental conditions including humidity, temperature, and occupancy levels, were not continuously monitored and may have influenced contamination variability.

Despite these limitations, the present study provides a comprehensive culture-based evaluation of gym surface microbial ecology and disinfection efficacy and advances understanding of microbial persistence dynamics within athletic built environments. The repeated-measures longitudinal design enabled detailed characterization of temporal contamination dynamics rarely captured in previous gym microbiology investigations. Integration of culture-based viability assessment with MALDI-TOF species identification improved taxonomic precision while maintaining direct relevance to viable microbial exposure risk. Furthermore, incorporation of ecological classification, persistence analysis, and real-world intervention assessment extended beyond purely descriptive contamination surveys. A major strength is the simultaneous evaluation of routine cleaning and H_2_O_2_ nebulization, providing translationally relevant evidence for environmental hygiene management in public fitness facilities. Inclusion of diverse equipment categories additionally enhanced ecological validity and enabled identification of device-specific contamination heterogeneity.

## 5. Conclusions

Gym equipment surfaces harbor dynamic, predominantly human-derived communities of recoverable viable bacteria characterized by rapid recolonization, persistence of skin-associated taxa, and substantial device-specific heterogeneity. Routine overnight cleaning demonstrated limited effectiveness, with 68% of environmental species no longer recovered after cleaning, whereas 93% of skin-associated species continued to be recovered, and achieved only a modest, non-significant reduction in recovered bacterial isolate counts. In contrast, the terminal decontamination procedure consisting of manual cleaning followed by H_2_O_2_ nebulization resulted in 88% of detected species no longer being recovered after treatment, with no Gram-negative species recovered in post-treatment cultures, whereas a small number of Gram-positive species continued to be recovered on certain devices. These findings suggest that fitness environments serve as microbial exchange ecosystems driven by continuous human contact, and that optimized hygiene management is likely to require a layered approach combining routine cleaning, appropriately controlled periodic no-touch H_2_O_2_ disinfection, targeted cleaning of high-contact equipment, and user hand hygiene. Because the terminal decontamination procedure was evaluated after a single application, these findings should be regarded as preliminary. The reproducibility, long-term effectiveness, potential for altered microbial susceptibility, and material compatibility associated with repeated H_2_O_2_-based decontamination in fitness environments require further investigation. In addition, the implementation of H_2_O_2_-based technologies should incorporate appropriate safety measures and validated operational protocols to maximize antimicrobial benefits while minimizing potential risks.

## Figures and Tables

**Table 1:** Device-specific microbial detection frequencies, diversity metrics, and temporal trends.

Gym Device ^1^	Recovered Isolates (n = 248)	Species (n = 61)	Families (n = 32)	*Staphylococcus*	Skin	Environment	Persistence ^2^	Statistical Trend ^3^
Functional trainer	26 (11%)	16	9	6 (23%)	19 (73%)	7 (27%)	36.4%	*p* = 0.22
Lat pulldown	20 (8%)	11	7	5 (25%)	16 (80%)	4 (20%)	22.2%	*p* = 0.29
Roman chair	19 (7%)	10	7	3 (16%)	14 (74%)	5 (26%)	36.4%	*p* = 0.79
Pulldown combo	18 (7%)	11	8	3 (17%)	13 (72%)	5 (28%)	44.4%	*p* = 0.89
Ab crunch machine	21 (9%)	10	8	6 (28%)	16 (76%)	5 (24%)	55.6%	*p* = 0.012 *
Leg extension	22 (9%)	15	10	9 (41%)	15 (68%)	7 (32%)	33.3%	*p* = 0.57
Reverse pec deck	22 (9%)	15	11	3 (14%)	12 (54%)	10 (46%)	36.4%	*p* = 0.07
45° leg press	19 (8%)	14	10	5 (26%)	12 (63%)	7 (37%)	33.3%	*p* = 0.14
Seated row machine	16 (6%)	10	9	2 (12%)	10 (62%)	6 (38%)	33.3%	*p* = 0.34
Arm ergometer	24 (10%)	11	6	10 (42%)	20 (83%)	4 (17%)	44.4%	*p* = 0.36
Upper body ergometer	19 (8%)	9	5	4 (21%)	17 (89%)	2 (11%)	36.4%	*p* = 0.006 *
Treadmill	22 (9%)	13	10	4 (18%)	14 (64%)	8 (36%)	36.4%	*p* = 0.15

^1^ Each device was sampled 15 times, with morning, midday, and evening collections conducted over five consecutive days. ^2^ Persistence (%) was calculated as the proportion of consecutive sampling intervals in which at least one bacterial species recovered from a device was also recovered during the immediately subsequent sampling interval. ^3^ The linear trend test over five consecutive days. * Significant increasing trend over five consecutive days.

## Data Availability

The original contributions presented in this study are included in the article/Supplementary Materials. Further inquiries can be directed to the corresponding authors.

## Supplementary Materials

The following supporting information can be downloaded at: https://www.mdpi.com/article/10.3390/microorganisms14081821/s1, Table S1: Distribution of bacterial families and species; Table S2: Paired isolates from the previous evening with the isolates in the next morning.
