# Supplementary material for: Effectiveness of Hydrogen Peroxide Nebulization Combined with Manual Cleaning in Reducing Surface-Associated Microorganisms in an Indoor Fitness Environment
Source: Microorganisms. 2026 Aug 18;14(8):1821. doi: 10.3390/microorganisms14081821 (PMC13515950; doi:10.3390/microorganisms14081821)
Supplement: Supplementary file 1 [file microorganisms-14-01821-s001.zip › microorganisms-4458750-supplementary.pdf]

Table S1: Distribution of bacterial families and species.

Bacterial Families	Species	Species Isolates	Family Isolates
Micrococcaceae	*Micrococcus luteus*	60	63
	Arthrobacter cumminsii	1	
	Kocuria rosea	1	
	Kocuria varians	1	
Staphylococcaceae	*Staphylococcus saprophyticus*	17	60
	Staphylococcus epidermidis	9	
	Staphylococcus xylosus	8	
	Staphylococcus cohnii	7	
	Staphylococcus hominis	7	
	Staphylococcus aureus	6	
	Staphylococcus warneri	4	
	Staphylococcus arlettae	2	
Moraxellaceae	*Acinetobacter lwoffii*	47	55
	Psychrobacter phenylpyruvicus	4	
	Acinetobacter johnsonii	2	
	Acinetobacter ursingii	2	
Pseudomonadaceae	*Pseudomonas stutzeri*	2	8
	Pseudomonas oryzihabitans	3	
	Pseudomonas luteola	1	
	Pseudomonas putida	1	
	Pseudomonas fluorescens	1	
Enterobacteriaceae	*Pantoea agglomerans*	6	7
	Serratia odorifera	1	
Bacillaceae	*Lysinibacillus fusiformis*	2	6
	Bacillus simplex	2	
	Bacillus	1	
	Lysinibacillus	1	
Aerococcaceae	*Acerococcus viridans*	5	5
Enterococcaceae	*Enterococcus faecalis*	2	4
	Enterococcus faecium	2	
Streptococcaceae	*Streptococcus constellatus*	2	4
	Lactococcus lactis	1	
	Streptococcus pneuminiae	1	
Vibrionaceae	*Vibrio vulnificus*	4	4
Xanthomonadaceae	*Stenotrophomonas maltophilia*	4	4
Brevibacteriaceae	*Brevibacterium casei*	1	2
	Cellulosimicrobium cellulans	1	
Brucellaceae	*Ochrobactrum anthropi*	2	2
Carnobacteriaceae	*Granulicatella adiacens*	1	2
	Carnobacterium divergens	1	
Caulobacteraceae	*Brevundimonas diminuta*	1	2
	Brevundimonas vesicularis	1	
Cellulomonadaceae	*Oerskovia turbata*	2	2
Dermacoccaceae	*Kytococcus sedentarius*	1	2
	Dermacoccus nishinomiyaensis	1	
Lactobacillaceae	*Alloiococcus otitis*	1	2
	Lactobacillus delbrueckii	1	
Microbacteriaceae	*Microbacterium arborescens*	1	2
	Leifsonia aquatica	1	
Alcaligenaceae	*Alcaligenes faecalis*	1	1
Clostridiaceae	*Clostridium sporogenes*	1	1
Coriobacteriaceae	*Collinsella aerofaciens*	1	1
Leuconostocaceae	*Leuconostoc mesenteroides*	1	1
Listeriaceae	*Listeria grayi*	1	1
Methylobacteriaceae	*Methylobacterium mesophilicum*	1	1
Neisseriaceae	*Eikenella corrodens*	1	1
Nocardiaceae	*Rhodococcus erythropolis*	1	1
Paenibacillaceae	*Paenibacillus pabuli*	1	1
Pasteurellaceae	*Haemophilus haemolyticus*	1	1
Propionibacteriaceae	*Propionibacterium acnes*	1	1
Rhizobiaceae	*Rhizobium radiobacter*	1	1

Table S2: Paired isolates from the previous evening with the isolates in the next morning.

Device ID	Monday evening/ Tuesday morning		Tuesday evening/ Wednesday morning		Wednesday evening/ Thursday morning		Thursday evening/ Friday morning	
**1**	*Bacillus simplex*		*S. hominis,<br>S. epidermidis*	*Acinetobacter lwoffii,<br>Micrococcus luteus*		*Pantoea agglomerans, Micrococcus luteus ,<br>Psychrobacter phenylpyruvicus*	*S. saprophyticus,<br>Micrococcus luteus*	
**2**	*Micrococcus luteus,<br>S. arlettae*	*Brevibacterium casei,<br>S. epidermidis*	*S. hominis,<br>Micrococcus luteus,<br>Acinetobacter ursingli*			*Acinetobacter johnsonii*		
**3**	*Enterococcus faecium*	*Micrococcus luteus*	*Acinetobacter lwoffii*	*Lysinibacillus fusiformis*		*Micrococcus luteus*	*Micrococcus luteus*	*S. cohnii, Acinetobacter lwoffii, Micrococcus luteus*
**4**	*Bacillus simplex,<br>Serratia odorifera,<br>S. warnerii,<br>Lactobacillus delbrueckii,<br>Micrococcus luteus*				*S. warnerii,<br>Micrococcus luteus*	*Psychrobacter phenylpyruvicus*	*Pantoea agglomerans,<br>S. xylosus, Aerococcus viridans, Micrococcus luteus*	
**5**		*S. saprophyticus*			*S. cohnii,<br>S. saprophyticus*	*S. aureus,<br>Acinetobacter lwoffii,<br>Micrococcus luteus*		*Acinetobacter lwoffii,<br>Pantoea agglomerans*
**6**	*Aerococcus viridans*	*Micrococcus luteus,<br>S. hominis*	*Micrococcus luteus,<br>S. epidermidis*		*S. arlettae ,<br>Kytococcus sedentarius,<br>Lysisnibacillus fusiformis*	*S. saprophyticus*		*S. xylosus*
**7**	*Micrococcus luteus , Alloiococcus otitis, Pseudomonas oryzihabitans,<br>S. epidermidis*	*Micrococcus luteus*	*Micrococcus luteus*	*Lysinibacillus fusiformis*	*Strepptococcus pneumoniae,<br>Vibrio vulnificus*	*Acinetobacter lwoffii, Pseudomonas stutzeri*		
**8**	*Granulcatella adiacens*			*Aerococcus viridans,<br>S. warneri, Micrococcus luteus*		*S. saprophyticus, Acinetobacter johnsonii*	*S. saprophyticus,<br>Micrococcus luteus*	*S. saprophyticus,<br>Enterococcus faecium,<br>Acinetobacter lwoffii*
**9**	*Oerskovia turbata,<br>Propionibacterium acnes*		*Acinetobacter lwoffii,<br>Micrococcus luteus*	*Acinetobacter lwoffii*			*Acinetobacter lwoffii*	
**10**	*Pseudomonas fluorescens,<br>Micrococcus luteus*		*Alcaligenes faecalis,<br>S. epidermidis*	*Acinetobacter lwoffii,<br>Listeria grayi*	*S. cohnii,<br>Micrococcus luteus,<br>S. cohnii*	*S. cohnii,<br>Acinetobacter lwoffii*	*Micrococcus luteus*	
**11**	*Micrococcus luteus*	*Micrococcus luteus*		*Acinetobacter lwoffii*		*S. xylosus,<br>Micrococcus luteus*	*S. hominis,<br>Micrococcus luteus*	*Acinetobacter ursingii ,<br>Arthrobacter cumminsii*
**12**	*Paenibacillus pabuli,<br>Stenotrophomonas maltophilia*		*Micrococcus luteus,<br>S. saprophyticus,<br>Acinetobacter lwoffii*	*Pseudomonas stutzeri*	*S. saprophyticus,<br>Micrococcus luteus*	*Acinetobacter lwoffii , Streptococcus constellatus,<br>Kocuria varians*	*Bevundimonas vesicularis,<br>Dermacoccus nishinomiyaensis*	*Acinetobacter lwoffii,<br>Pantonea agglomerans*
Total number Isolates	**22**	**8**	**16**	**12**	**14**	**21**	**15**	**12**

Gym devices: 1. Functional trainer, 2. Lat pulldown, 3. Roman chair, 4. Pulldown combo, 5. Ab crunch machine, 6. Leg extension, 7. Reverse pec deck, 8. 45° leg press, 9. Seated row machine, 10. Arm ergometer, 11. Upper body ergometer, 12. Treadmill.
